# Occupational Health Hazards and Management Practices Among Nurses in Public Healthcare Facilities in Four Sub‐Sahara African Countries: A Scoping Review

**DOI:** 10.1155/jonm/8510130

**Published:** 2026-09-07

**Authors:** Kristofina Tsenaye Nakatana, Hans Justus Amukugo, Anna Leonard

**Affiliations:** ^1^ Department of General Nursing, Faculty of Health Sciences and Veterinary Medicine, University of Namibia, Windhoek, Khomas, Namibia, unam.edu.na; ^2^ Research Support Services, Information Learning Resource Centre, Library Services, University of Namibia, Windhoek, Khomas, Namibia, unam.edu.na

**Keywords:** nurses, occupational health hazards, scoping review, sub-Sahara Africa

## Abstract

**Background:**

Nurses in sub‐Sahara Africa are disproportionately exposed to occupational health hazards. These risks are intensified by persistent shortages of resources, inadequate training, weak reporting systems and fragile safety infrastructures, all of which undermine nurses’ well‐being and compromise the quality of healthcare delivery.

**Purpose:**

The aim of this study was to map the existing evidence on practices for managing occupational health hazards among nurses in public healthcare facilities in four sub‐Sahara African countries: Ghana, South Africa, Ethiopia and Uganda.

**Methods:**

A scoping review was conducted in accordance with Arksey and O’Malley’s (2005) five‐stage methodological framework and reported following the PRISMA‐ScR checklist. Comprehensive research was performed across PubMed, ScienceDirect, Cochrane, SpringerLink and Wiley, supplemented with grey literature and WHO/ILO resources. Fourteen studies published between 2017 and 2024 met the inclusion criteria.

**Results:**

The study identified four overarching themes: occupational health hazards in healthcare facilities, nursing workforce challenges, nursing health and lifestyle, and emergency preparedness. These themes collectively demonstrate the wide spectrum of risks to which nurses are exposed, the systemic deficiencies that heighten their vulnerability, the adverse consequences for their health and well‐being, and the insufficient readiness of health systems to respond effectively to public health crises.

**Conclusion:**

The findings confirm that nurses in sub‐Sahara Africa are exposed to a variety of occupational hazards that compromise their safety and professional sustainability. Although interventions such as PPE provision, ergonomic training and infection control measures exist, their implementation remains fragmented. Strengthening occupational health and safety policies, enhancing resource allocation, institutionalizing continuous professional training and developing resilient preparedness frameworks are essential to protect nurses and reinforce healthcare system resilience in the region.

**Implications for Nursing Management:**

Nursing leadership must prioritize occupational health and safety through adequate staffing, reliable PPE provision, continuous training and effective reporting systems to protect nurses and strengthen healthcare system resilience.

## 1. Introduction

Nurses constitute the largest group of healthcare professionals worldwide and are the backbone of healthcare systems. In 2020, the World Health Organization (WHO) estimated the global nursing workforce at 27.9 million, representing nearly half of the entire healthcare workforce [1]. Despite this critical role, there is a persistent global shortage of nurses, particularly in low‐ and middle‐income countries, where health systems are under severe strain [1, 2]. This shortage not only increases the workload for existing staff but also elevates their risk of exposure to occupational hazards.

Occupational health hazards among nurses include biological, psychological, ergonomic, physical and mechanical risks, all of which threaten their safety, productivity and the quality of care delivered to patients. According to the International Labour Organization (ILO), work‐related diseases accounted for 2.58 million deaths (89%) of all occupationally related deaths globally in 2019, while injuries accounted for the remaining 11% [3]. Nurses are disproportionately affected because they provide frontline care, often with prolonged patient contact and exposure to hazardous environments [4].

The study focuses exclusively on nurses working in public healthcare facilities rather than private and nongovernmental healthcare facilities because public healthcare settings are often characterized by resource constraints, inadequate infrastructure and high patient workloads, factors that may increase nurses’ exposure to occupational health hazards [3, 4]. In contrast, private and nongovernmental healthcare facilities generally have better access to resources, more adequate infrastructure and lower patient‐to‐nurse ratios, which may contribute to safer working environments and reduced exposure to occupational health hazards among nurses [5].

In sub‐Sahara Africa, nurses face an even heavier burden due to systemic resource shortages, inadequate occupational health infrastructure and weak policy enforcement. Communicable diseases pose a significant challenge: The WHO regional report indicates that more than 30% of all work‐related mortality in the African region is linked to communicable diseases, compared to less than 5% in high‐income countries [5]. Needlestick injuries are alarmingly prevalent, with studies reporting rates ranging from 42% to 70% among African nurses, largely due to limited personal protective equipment (PPE), unsafe sharps disposal practices and high patient volumes [6].

Beyond biological hazards, musculoskeletal disorders (MSDs) are common among African nurses, with 44%–78% reporting musculoskeletal pain and low back pain affecting more than half (54%–64%) [7]. Poor ergonomic design, inadequate equipment and frequent manual patient lifting contribute significantly to these conditions. Workplace violence is another occupational challenge: The prevalence of verbal abuse and physical assault against nurses in African healthcare facilities ranges from 40% to 70% [8]. Psychological hazards are equally widespread, with burnout rates estimated between 50% and 70%, exacerbated by understaffing, long working hours and poor managerial support [9].

The COVID‐19 pandemic highlighted the lack of emergency preparedness among nurses in the region. Surveys in Kenya, Ghana and South Africa showed that over 60% of nurses felt inadequately prepared for public health crises, citing PPE shortages, poor infection prevention measures and unclear protocols [10]. Reporting of occupational injuries remains low fewer than 35% of nurses formally report incidents in countries such as Namibia, Ethiopia and Ghana due to fear of reprisal and inefficient reporting systems [11].

Despite the existence of global occupational‐health frameworks such as the WHO healthy workplace framework and the CDC workplace health model, their adoption and implementation in sub‐Sahara African nursing practice remain inconsistent. This underscores the urgent need to map existing evidence on occupational‐health hazard management practices among nurses in the region. The study aligns with SDG 3 (good health and well‐being) and SDG 8 (decent work and economic growth) [12]. Scoping reviews, therefore, play a critical role in mapping available evidence, identifying best practices and highlighting gaps that hinder effective policy formulation. The aim of this study was to map existing evidence on practices for managing occupational‐health hazards among nurses in state health facilities in sub‐Sahara Africa.

### 1.1. Study Area

In sub‐Sahara Africa, studies conducted in Ghana, Ethiopia, South Africa and Uganda have shown that nurses are frequently exposed to occupational hazards within health facilities ranging from government hospitals and psychiatric institutions to regional and specialized referral hospitals. Nurses form the backbone of the health workforce across these countries, often serving as the first point of contact for patients in hospitals, health centres, clinics and dispensaries. However, these facilities are often under‐resourced, overcrowded and unevenly distributed, which, combined with high patient‐to‐nurse ratios, places nurses at significant risk of biological, physical and psychosocial hazards.

**FIGURE 1:**
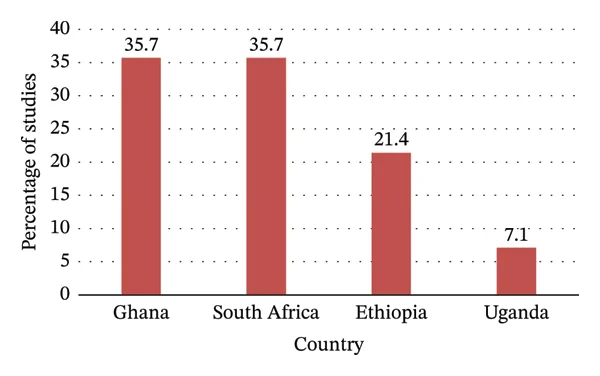
Distribution of studies conducted in sub‐Saharan Africa.

As illustrated in Figure 1, the distribution of studies in the reviewed literature shows that Ghana (35.7%) and South Africa (35.7%) accounted for the largest share of research, followed by Ethiopia (21.4%) and Uganda (7.1%). This distribution indicates that while occupational hazards among nurses are widely recognized in Ghana and South Africa, other countries such as Uganda remain underrepresented, despite facing similar risks. In Ghana, studies documented high levels of stress, burnout and hazard exposure among nurses, with infection risks, manual patient lifting, poor supervision and weak reporting mechanisms identified as major challenges. Enablers included targeted training, engaged leadership and policy enforcement. In Ethiopia, facility‐based studies revealed a 73.8% prevalence of MSDs, a 36.2% prevalence of needlestick injuries and widespread workplace violence in surgical and psychiatric wards. Lack of ergonomic training and poor safety systems were common obstacles, while ergonomic awareness and reporting systems were suggested as solutions. Specifically, the 14 included studies originated from four countries only: Ghana (5 studies, 35.7%), South Africa (5 studies, 35.7%), Ethiopia (3 studies, 21.4%) and Uganda (1 study, 7.1%).

In South Africa, research from psychiatric hospitals, public hospitals and mental health institutions reported workplace violence, inadequate PPE, heavy workloads, emotional strain and fear of infection among nurses, especially in tuberculosis (TB) units. Workplace health promotion programmes, peer support and improved safety measures were identified as enablers. In Uganda, a study in Northern hospitals found nearly half of nurses experienced high burnout levels, associated with heavy workloads, poor management and weak social support. Suggested interventions included healthier diets, peer support and adjusted working hours.

Collectively, these studies highlight that nurses in sub‐Sahara Africa face biological hazards (HIV, TB and needlestick injuries), physical hazards (violence, musculoskeletal strain and ergonomic risks) and psychosocial hazards (stress, burnout and poor lifestyle behaviours). Weak reporting systems, shortages of resources and lack of safety training worsen these risks, though practical enablers such as PPE provision, workplace wellness programmes and occupational health training offer pathways for improvement.

## 2. Methods

This review was conducted using the scoping review methodology outlined by Arksey and O’Malley [1], expanded by Levac et al. [2] and further refined by Peters et al. [3]. The study was reported in accordance with the Preferred Reporting Items for Systematic Reviews and Meta‐Analyses extension for Scoping Reviews (PRISMA‐ScR) guidelines [4]. This review was guided by the research question: “*What is the existing evidence on occupational health hazards experienced by nurses and the management practices implemented in public healthcare facilities across Sub-Saharan Africa?”* A formal methodological quality appraisal of the included studies was not conducted. This review followed the Joanna Briggs Institute (JBI) methodology for scoping reviews, which is primarily intended to map the breadth and nature of available evidence rather than to assess the methodological quality or risk of bias of individual studies. Consistent with JBI guidance and the objectives of scoping reviews, the focus of this review was on identifying, describing and synthesizing existing evidence on occupational health hazards and management practices among nurses in public healthcare facilities in selected sub‐Sahara African countries. The protocol was registered with the Open Science Framework (OSF) and is available at https://osf.io/confirm/external/swa6v/dIG8crPJE71d8piNTRtGxZCNyCq2cg/?destination=dashboard.

### 2.1. Search Strategy

The Rayyan systematic review software was employed to facilitate the screening of titles and abstracts using Medical Subject Headings (MeSH) terms and relevant keywords. The focus was on identifying literature related to practices in the management of occupational health hazards among nurses within public healthcare facilities in sub‐Sahara Africa. The search terms, along with their associated subject headings and MeSH terms, were developed in consultation with an academic librarian to ensure a comprehensive strategy. The search strategy incorporated the following MeSH terms and Boolean operators: “Practice AND occupational health hazard” OR “occupational injury” OR “workplace injury” AND “management” OR “control” AND “nurse” OR “midwife” OR “healthcare worker^∗^” AND “hospital^∗^” OR “healthcare facility^∗^” AND “Sub‐Sahara Africa^∗^.” In addition, data organization, cross‐checking and duplicate removal were conducted using online citation management software. After the records were screened and the eligibility criteria were applied, only a smaller number of relevant articles remained.

Using these keywords, searches were conducted in the following electronic databases: ScienceDirect, EBSCOhost, Cochrane, PubMed, SpringerLink, Wiley and HINARI. These databases were selected because they are widely recognized as comprehensive and reliable sources of evidence in the health sciences. To capture unpublished and grey literature, additional searches were conducted in Grey Literature Reports, OpenGrey and PubMed Conference Proceedings. The grey literature search identified 12 records from OpenGrey and 6 records from conference proceedings. After screening, no studies from conference proceedings met the final inclusion criteria, while 2 records from OpenGrey were considered during full‐text review but were excluded because they did not meet the eligibility criteria. Table 1 illustrates the types of MeSH terms used in each database, as some terms were modified to suit the specific search strategies of each database.

**TABLE 1:** The types of MeSH terms used in each database.

Data bases	Mesh terms used	Number of articles obtained
Ebsco Host	“Practice AND management AND Occupational exposure occupational hazards OR occupational risks OR occupational diseases OR occupational injuries OR occupational accidents OR Health Personnel OR health care workers OR health professionals OR AND nurse OR healthcare OR midwives AND state health care facilities OR public health facility AND Sub‐Saharan Africa”	*n* = 32
Cochrane	practice AND management AND Occupational exposure OR occupational hazards OR occupational risks OR occupational diseases OR occupational injuries OR occupational accidents AND nurse^∗^healthcare OR midwives AND state health care facilities OR public health facilities AND Sub‐Saharan Africa	*n* = 20
Science direct	practice AND occupational health hazard AND management AND nurse AND hospital OR AND Sub‐Saharan Africa	*n* = 46
Hinary	practice regarding management of occupational health hazards among nurses in state health care facility in Sub‐Sahara Africa	*n* = 21
Springer link	practice AND occupational health hazard AND management AND nurse AND hospital OR AND sub‐Saharan Africa	*n* = 76
Wiley	“Practice AND occupational hazards nurse OR healthcare OR midwives AND state health care facilities OR public health facilities AND Sub‐Saharan Africa”	*n* = 44
PubMed	Best practice AND management AND Occupational exposure OR occupational hazards OR occupational risks OR occupational diseases OR occupational injuries OR occupational accidents AND health care workers OR nurse^∗^healthcare OR midwives AND state health care facilities OR public health facilities AND Sub‐Saharan Africa	*n* = 141
Total bases = 7		*n* = 380

### 2.2. Study Selection

Studies published between 2017 and 2024 in sub‐Sahara Africa focussing on nurses were included. Eligible designs encompassed both quantitative and qualitative studies, as well as text and opinion papers. Searches were conducted independently by two reviewers Kristofina Tsenaye Nakatana (KTN) and Anna Leonard (AL), and any disagreements were resolved by a third reviewer Hans Justus Amukugo (HJA). The screening and inclusion process was further validated by expert librarians from the University of Namibia Anna Leonard (AL). Duplicates were removed, reference lists manually searched, and only a limited number of studies met the final inclusion criteria. Manual searches of the reference list of the included studies were also conducted.

#### 2.2.1. Charting of the Data

Data extraction was independently conducted by two reviewers, Kristofina Tsenaye Nakatana (KTN) and Anna Leonard (AL), using a standardized data‐charting form for the final 14 included studies. Any disagreements were resolved through discussion with Hans Justus Amukugo (HJA).

#### 2.2.2. Collating and Summarizing the Results

Study characteristics including author, year, country, topic, study population, study design, methods, findings together with source information and key outcomes, are presented in figures. A thematic analysis was then conducted, and studies were grouped into occupational hazard categories and types of exposure according to the WHO classification of occupational hazards in nursing [13]. This approach facilitated the identification of dominant research areas, key variables and existing gaps. The overall findings are presented as a narrative review.

#### 2.2.3. Results

The database searches identified 380 articles. After the removal of 79 duplicate records, 301 unique records remained for title and abstract screening. During this stage, 202 records were excluded because they did not meet the inclusion criteria, primarily due to involving non‐nursing populations or being conducted outside sub‐Sahara Africa. The remaining 99 articles were retrieved and assessed for full‐text eligibility, with no reports unavailable for retrieval. Upon full‐text review, 85 articles were excluded because they did not provide relevant information to address the review objective or focused on maternal health rather than occupational health hazards among nurses. Consequently, 14 studies satisfied all eligibility criteria and were included in the qualitative synthesis.

**FIGURE 2:**
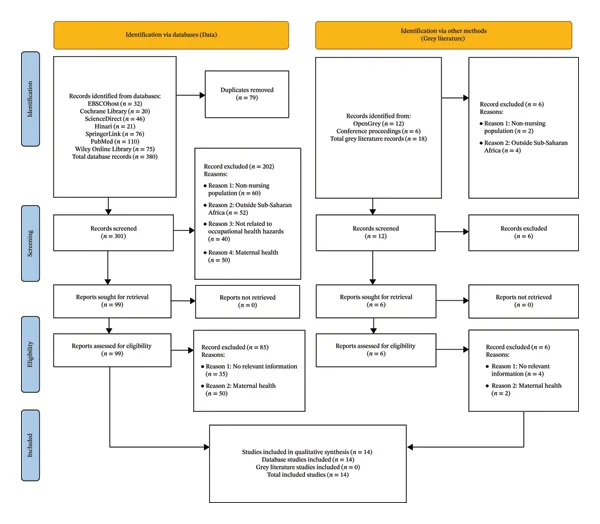
PRISMA study selection flowchart.

In addition, 18 database search records were identified through grey literature sources, including open grey and conference proceedings. Moreover, six records were excluded during the initial screening stage because they involved non‐nursing populations and some conducted outside sub‐Sahara Africa. The remaining six reports were retrieved and assessed for eligibility. However, all six reports were subsequently excluded following full‐text review because they either contained no useful information relevant to the review objectives or focused on maternal health. Consequently, no grey literature studies met the eligibility criteria for inclusion in the final qualitative synthesis. The study selection process and reasons for exclusion at each stage are presented in Figure 2.

**FIGURE 3:**
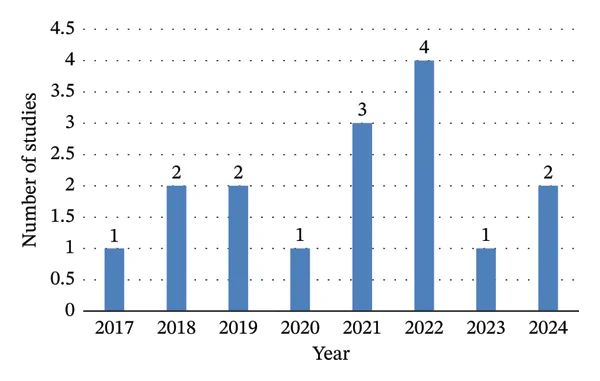
Trends of study by year of publication.

Figure 3 presents the distribution of included studies by year of publication. The included studies were published between 2017 and 2024, with eligibility restricted to studies conducted in sub‐Sahara Africa and focussing on nurses.

**FIGURE 4:**
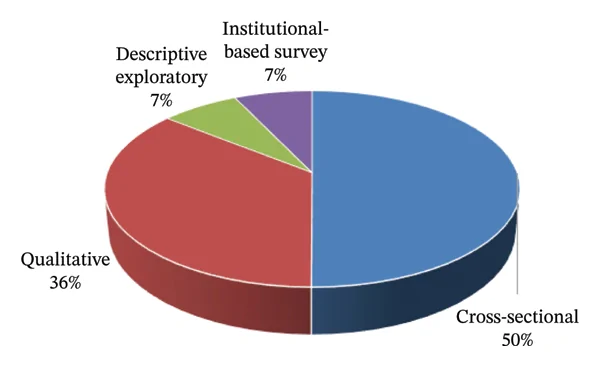
Study designs distribution.

Figure 4 presents a pie chart illustrating the distribution of study designs among the included studies.

The distribution of study designs among the 14 included studies showed that cross‐sectional studies were the most common design, accounting for 50.0% (*n* = 7) of the included studies. Qualitative studies represented 35.7% (*n* = 5) of the studies. Descriptive exploratory studies and institutional‐based survey studies each accounted for 7.1% (*n* = 1). This distribution indicates that the available evidence on occupational health hazards among nurses in sub‐Sahara Africa is predominantly derived from cross‐sectional and qualitative research designs.

#### 2.2.4. Thematic Findings

A thematic analysis of the included studies revealed four primary themes and fourteen subthemes. These findings are systematically presented in Table 2, which summarizes the identified thematic structure.

**TABLE 2:** Summary of the themes and subthemes that emerged from the thematic analysis of the research articles.

Main theme	Subtheme
1. Occupational Health Hazards in Health Facilities	1.1 Biological
	1.2 Psychological
	1.3 Ergonomic
	1.4 Physical
	1.5 Mechanical

2. Nursing Workforce Challenges and Management	2.1 Lack of Resources
	2.2 High Workload
	2.3 Lack of Training
	2.4 Inadequate Safety Measures
	2.5 Lack of compensation
	2.6 Inadequate Reporting Systems

3. Health and Lifestyle of Nurses	3.1 Lack of Lifestyle and Health Choice
	3.2 Impact of Work on Health and Well‐being

4. Emergency Preparedness	4.1 Preparedness for Public Health Crises

## 3. Discussion

This scoping review synthesized evidence on occupational health hazards and management practices among nurses working in public healthcare facilities in sub‐Sahara Africa. The findings demonstrated that nurses are exposed to multiple occupational hazards, including biological, psychological, ergonomic, physical and mechanical hazards, which negatively affect their health, well‐being and professional performance. Workforce shortages, inadequate resources, poor occupational health and safety (OHS) measures, weak reporting systems and insufficient emergency preparedness further increase nurses’ vulnerability and compromise the quality of healthcare delivery [3, 5, 10, 11].

The findings of this review can be interpreted using the Donabedian model of structure, process and outcomes, which provides a framework for understanding occupational health management systems in healthcare settings [1, 2]. Structural factors such as inadequate staffing, shortages of PPE, weak occupational health policies and poor infrastructure influence healthcare processes, including infection prevention practices, occupational health training, incident reporting and emergency preparedness [1, 2, 10, 12]. Deficiencies in these processes subsequently contribute to adverse outcomes such as occupational injuries, burnout, absenteeism, reduced quality of care and poor nurse well‐being [5–8]. The integration of the Donabedian model into the discussion strengthened the interpretation of healthcare system weaknesses and supported the identification of practical management interventions to improve OHS among nurses.

### 3.1. Occupational Health Hazards in Healthcare Facilities

The review identified a wide range of occupational health hazards experienced by nurses in healthcare facilities across sub‐Sahara Africa [3–9].

### 3.2. Biological Hazards

Needlestick injuries and exposure to infectious diseases such as TB, hepatitis B virus (HBV), hepatitis C virus (HCV), and human immunodeficiency virus (HIV) were among the most frequently reported occupational hazards [3, 4]. Poor ventilation systems, inadequate PPE, unsafe disposal of sharps and weak infection prevention measures contributed to increased exposure risks [3, 4, 12]. Healthcare managers should strengthen infection prevention and control programmes through continuous PPE provision, vaccination programmes, safe sharps disposal systems, regular occupational health training and strict enforcement of standard precautions to reduce biological hazards among nurses [3, 4, 12].

### 3.3. Psychological Hazards

Psychological hazards such as occupational stress, burnout, workplace violence, anxiety and emotional exhaustion were highly prevalent among nurses [5–8]. Excessive workloads, staff shortages, long working hours and poor managerial support significantly contributed to burnout and stress [5–8]. Workplace violence also contributed to absenteeism, low morale and reduced job satisfaction [7]. Practical management strategies such as workplace wellness programmes, staff counselling services, stress‐management interventions, supportive supervision, violence‐prevention policies and adequate staffing should be implemented to improve nurses’ psychological well‐being and reduce burnout [5–8].

### 3.4. Ergonomic Hazards

MSDs were commonly reported among nurses due to prolonged standing, repetitive movements, heavy lifting and poor working postures [9]. Heavy workloads and inadequate ergonomic equipment further contributed to physical strain and fatigue [9].

Healthcare institutions should implement ergonomic interventions including provision of patient lifting devices, ergonomic workplace redesign, safe patient‐handling training and adequate staffing to reduce musculoskeletal injuries among nurses [9].

### 3.5. Physical Hazards

Nurses experienced physical hazards such as slips, falls, workplace violence and patient‐handling injuries [7, 9]. Poor infrastructure, overcrowded wards and unsafe working environments increased occupational injury risks [7, 9].

Healthcare managers should strengthen workplace safety through environmental modifications, improved security systems, routine safety inspections and implementation of violence‐prevention protocols to reduce physical hazards in healthcare facilities [7, 9].

### 3.6. Mechanical Hazards

Mechanical hazards associated with poorly maintained medical equipment and unsafe healthcare environments were identified [10, 11]. Inadequate servicing of medical devices, lack of maintenance and weak safety protocols increased the risk of occupational injuries and compromised patient safety [10, 11]. Regular equipment maintenance schedules, strengthening biomedical engineering services and enforcement of occupational safety standards are necessary to minimize mechanical hazards and improve patient and nurse safety [10, 11].

Overall, occupational health hazards remain a major challenge for nurses in sub‐Sahara Africa. Interventions such as adequate PPE provision, ergonomic workplace redesign, occupational health training, violence‐prevention strategies and strengthened infection prevention measures are necessary to improve nurse safety and well‐being [3, 7, 9, 12].

### 3.7. Nursing Workforce Challenges and Management

The review found that workforce‐related challenges significantly contributed to occupational health hazards and reduced healthcare quality [10, 11, 12, 14].

### 3.8. Lack of Resources

Shortages of healthcare workers, inadequate medical supplies, insufficient PPE and poor infrastructure forced nurses to work under unsafe conditions [10, 11]. Addressing these challenges requires improved healthcare funding, adequate resource allocation, strengthening supply chain systems and investment in healthcare infrastructure and staffing to improve OHS conditions [10, 11].

### 3.9. High Workload

Excessive workloads and long working hours contributed to fatigue, stress, burnout and clinical errors [5, 6]. Overcrowded healthcare facilities and inadequate staffing further increased nurses’ exposure to occupational injuries and infectious diseases [3, 10]. Management interventions such as recruitment of additional nursing staff, workload redistribution, flexible scheduling and implementation of staff retention strategies are essential to reduce excessive workloads and burnout among nurses [5, 6, 10].

### 3.10. Lack of Training

Several studies reported inadequate OHS training among nurses [12, 14]. Limited emergency preparedness training further increased nurses’ vulnerability during public health crises [14]. Healthcare institutions should institutionalize continuous occupational health education programmes, emergency preparedness training, infection prevention workshops and professional development initiatives to strengthen nurses’ competencies and occupational safety practices [12, 14].

### 3.11. Inadequate Safety Measures

Weak infection prevention practices, inconsistent PPE supply, poor workplace security and absence of violence‐prevention protocols exposed nurses to avoidable occupational hazards [3, 7, 12]. Strengthening occupational health policies, ensuring continuous PPE availability, improving infection prevention practices and implementing workplace violence prevention strategies are essential to improve occupational safety and reduce workplace injuries [3, 7, 12].

### 3.12. Lack of Compensation and Support

The absence of occupational health insurance, hazard allowances and psychological support services reduced nurse motivation and increased turnover intentions [5, 10]. Provision of hazard allowances, occupational health insurance, employee wellness programmes and psychological support services may improve staff motivation, retention and overall well‐being among nurses [5, 10].

### 3.13. Inadequate Reporting Systems

Fear of victimization and weak institutional reporting procedures contributed to underreporting of occupational incidents and workplace violence [7, 12]. Inadequate reporting systems contributed to the underreporting of occupational incidents, limiting opportunities for institutional learning and improvements in workplace safety [7]. Healthcare institutions should establish transparent, confidential, and nonpunitive incident reporting systems to encourage reporting and strengthen occupational safety monitoring and management [7, 12]. Addressing nursing workforce challenges requires stronger occupational health policies, improved staffing levels, continuous occupational health training, transparent reporting systems and adequate allocation of healthcare resources to strengthen nurse safety and healthcare service delivery [10, 11, 12, 14].

### 3.14. Health and Lifestyle of Nurses

The demanding healthcare work environment negatively affected nurses’ physical and psychological health [5, 6, 15].

### 3.15. Lifestyle and Health Challenges

Long working hours, fatigue and occupational stress limited opportunities for healthy lifestyle practices such as exercise, healthy eating and adequate rest [5, 14]. Nurses reported concerns related to obesity, hypertension, stress and noncommunicable diseases associated with demanding work schedules [14]. Healthcare institutions should implement workplace wellness programmes, promote healthy lifestyle initiatives, encourage physical activity, and provide adequate rest periods to support nurses’ overall health and well‐being [14].

### 3.16. Impact on Health and Well‐Being

Occupational stress, burnout, MSDs and exposure to infectious diseases negatively affected nurses’ health and well‐being [3, 5, 6, 9]. These conditions contributed to absenteeism, emotional exhaustion, reduced productivity and increased staff turnover [5, 6]. Stress‐management programmes, occupational health services, mental health support systems, and ergonomic interventions are necessary to improve nurses’ health outcomes, productivity and work performance [5, 9, 14].

### 3.17. Emergency Preparedness and Crisis Management

The review identified inadequate emergency preparedness and crisis management systems in healthcare facilities across sub‐Sahara Africa [12, 14].

### 3.18. Preparedness for Public Health Crises

Insufficient training, unclear emergency response protocols, inadequate PPE and limited healthcare resources compromised nurses’ ability to respond effectively during public health emergencies such as the COVID‐19 pandemic [12]. These deficiencies increased occupational risks and reduced the effectiveness of healthcare responses during crises [15]. The Donabedian model explains how weaknesses in healthcare structures and processes contribute to poor occupational health outcomes during crises [15]. Weak staffing systems, inadequate resources, poor infrastructure and ineffective preparedness processes increase occupational risks and compromise healthcare delivery during emergencies [1, 2]. To strengthen emergency preparedness, healthcare institutions should develop comprehensive emergency preparedness plans, conduct regular simulation drills, maintain emergency PPE stockpiles, strengthen infection prevention systems and provide continuous disaster preparedness training for nurses [12, 14]. Strengthening healthcare structures, improving emergency preparedness processes and monitoring occupational health outcomes are essential for developing resilient healthcare systems in sub‐Sahara Africa [1, 2].

## 4. Application of the Donabedian Structure–Process–Outcome (SPO) Framework to Occupational Health Hazard Management

**FIGURE 5:**
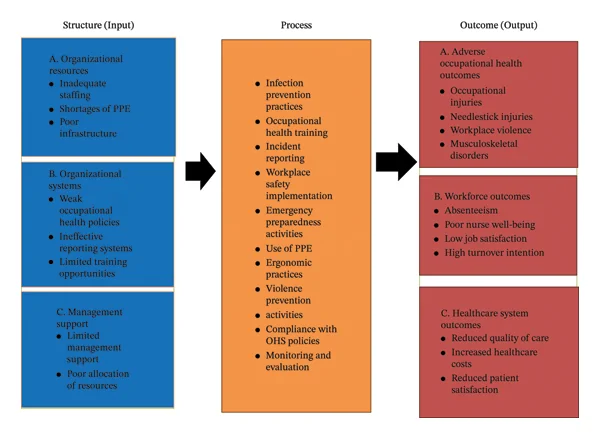
Donabedian conceptual framework.

The findings of this review can be interpreted using the Donabedian SPO model, originally developed by Avedis Donabedian in 1966 as a framework for evaluating the quality of healthcare services. According to this model, healthcare quality can be assessed through three interconnected components: structure, process, and outcomes. Structure refers to the organizational resources, and conditions under which care is delivered [15] process refers to activities and practices undertaken in service delivery; and outcomes refer to the effects of these activities on health and service performance [15]. In the context of this review, structural factors such as inadequate staffing, shortages of PPE, weak occupational health policies, limited training opportunities, poor infrastructure and ineffective reporting systems influenced occupational health management processes, including infection prevention practices, occupational health training, incident reporting, workplace safety implementation and emergency preparedness activities. These process‐related deficiencies subsequently contributed to adverse outcomes such as occupational injuries, needlestick injuries, workplace violence, MSDs, burnout, absenteeism, poor nurse well‐being and reduced quality of healthcare delivery [16]. The Donabedian framework is particularly relevant to occupational health hazard management because it enables a systematic examination of how organizational factors influence safety practices and, ultimately, occupational health outcomes among nurses [15]. Figure 5: illustrates how the findings of this review were mapped onto the SPO framework.

## 5. Conclusion

This scoping review revealed that nurses in sub‐Sahara Africa are exposed to multiple occupational health hazards, including biological, psychological, ergonomic, physical and mechanical risks [3–11]. The findings showed that inadequate infection prevention measures, poor workplace safety systems, insufficient PPE, lack of ergonomic support, workplace violence and poorly maintained equipment continue to compromise nurses’ health, safety and professional well‐being [3, 4, 7, 9–12]. These occupational hazards negatively affect healthcare delivery, increase absenteeism and burnout, and weaken healthcare system resilience [5–8].

The review further demonstrated that workforce‐related challenges such as staff shortages, excessive workloads, inadequate occupational health training, poor compensation and weak reporting systems significantly increase nurses’ vulnerability to occupational hazards [5, 6, 10, 11, 12, 14]. Inadequate emergency preparedness, limited disaster‐response training and insufficient institutional support during public health crises further expose nurses to occupational risks and compromise effective healthcare responses [12, 14].

In addition, the findings highlighted that demanding work environments, long working hours, occupational stress and poor work‐life balance contribute to chronic health conditions, burnout, emotional exhaustion and reduced productivity among nurses [5, 6, 14]. These challenges emphasize the need for stronger occupational health management systems and supportive workplace environments to improve nurses’ well‐being and healthcare outcomes.

Overall, this review highlights the urgent need for holistic and evidence‐based interventions that address both immediate occupational risks and long‐term structural barriers within healthcare systems. Strengthening OHS policies, improving resource allocation, ensuring continuous occupational health training, implementing ergonomic and workplace wellness interventions, strengthening reporting systems and improving emergency preparedness are critical to safeguarding nurses’ well‐being and strengthening healthcare system resilience in sub‐Sahara Africa [1, 2, 10, 11, 12, 14]. These interventions are also essential for supporting the achievement of Sustainable Development Goal 3 (Good Health and Well‐Being) and Sustainable Development Goal 8 (Decent Work and Economic Growth).

## 6. Recommendations

Based on the findings of this review, governments and healthcare institutions in sub‐Sahara Africa should strengthen and enforce OHS policies to improve workplace safety for nurses. Greater investment is needed to ensure adequate provision of PPE, safe medical equipment, sufficient staffing and improved healthcare infrastructure to reduce occupational risks and workload pressures.

Healthcare institutions should implement continuous OHS training programmes focussing on infection prevention and control, ergonomic practices, workplace violence prevention, incident reporting and emergency preparedness. Regular simulation exercises and disaster preparedness training should also be institutionalized to improve nurses’ readiness during public health emergencies.

Healthcare managers should establish transparent, confidential and nonpunitive reporting systems to improve monitoring and management of occupational incidents, workplace injuries and violence against nurses. Strengthening occupational health surveillance systems will support evidence‐based decision‐making and continuous workplace safety improvement.

In addition, healthcare institutions should prioritize nurses’ physical and psychological well‐being through implementation of workplace wellness programmes, mental health support services, stress‐management interventions, adequate rest periods and fair compensation mechanisms, including occupational health insurance and hazard allowances. Finally, stronger collaboration between governments, healthcare institutions, academic institutions, nongovernmental organizations and professional nursing bodies is essential to strengthen occupational health systems, improve healthcare resilience and promote safer and healthier working environments for nurses across sub‐Sahara Africa.

## Author Contributions

Kristofina Tsenaye Nakatana (KTN) conceptualized the study, developed the search strategy, screened and extracted the data, and drafted the manuscript. Kristofina Tsenaye Nakatana (KTN) also served as the corresponding author. Anna Leonard (AL) contributed to data screening, analysis and editing of the manuscript, while Hans Justus Amukugo (HJA) provided critical revisions and input on the thematic analysis.

## Funding

The study has not been funded.

## Disclosure

This scoping review included only 14 studies conducted across four sub‐Sahara African countries, namely, Ghana, South Africa, Ethiopia and Uganda. Accordingly, the findings should be interpreted with caution, as they may not adequately reflect the diverse occupational health realities, healthcare systems, policy environments and resource contexts that characterize the broader sub‐Sahara African region. Furthermore, methodological variations across the included studies, together with the limited availability of occupational health research from several African countries, may have constrained the breadth and comprehensiveness of the evidence synthesized in this review.

Moreover, a formal methodological quality appraisal of the included studies was not undertaken. This approach is consistent with the Joanna Briggs Institute (JBI) methodology for scoping reviews, which emphasizes the identification, mapping and synthesis of available evidence rather than the critical assessment of methodological quality or risk of bias in individual studies. Consequently, the methodological rigour and overall strength of the included evidence could not be formally established.

All authors read and approved the final version of the manuscript.

## Ethics Statement

The authors have nothing to report.

## Consent

The authors have nothing to report.

## Conflicts of Interest

The authors declare no conflicts of interest.

## Supporting Information

Additional supporting information can be found online in the Supporting Information section.

## Supporting information


**Supporting Information** PRISMA‐ScR‐Fillable‐Checklist.

## Data Availability

The data supporting the findings of this study are available upon request from the corresponding author. The data are not publicly available because of privacy and ethical restrictions on data sharing.
